# A rare case report of endobronchial neurofibroma treated with transbronchial endoscopic resection and literature review

**DOI:** 10.1097/MD.0000000000039730

**Published:** 2024-09-20

**Authors:** Xing Chen, Shanlu Yu, Jian Sun, Yefeng Chen, Chunyi Zhang, Hua Wang, Min Xiang, Shuying Liu

**Affiliations:** a Department of Respiratory Medicine, Shaoxing People’s Hospital (Shaoxing Hospital, Zhejiang University School of Medicine), Shaoxing, Zhejiang Province, China; b Department of Pathology, Shaoxing People’s Hospital (Shaoxing Hospital, Zhejiang University School of Medicine), Shaoxing, Zhejiang Province, China; c Department of Cardiology, Shaoxing People’s Hospital (Shaoxing Hospital, Zhejiang University School of Medicine), Zhejiang Province, China.

**Keywords:** electrical snaring, endobronchial neurofibroma, laser coagulation, recurrence, transbronchial endoscopic resection

## Abstract

**Rationale:**

Endobronchial neurofibroma is an extremely rare neoplastic disease. The majority of endobronchial neurofibroma are symptomatic, but nonspecific. The treatment of endobronchial neurofibroma is controversial that surgery is previously considered to be the main option. With the development of bronchoscopic intervention, most endobronchial neurofibroma can be treated with transbronchial endoscopic resection with few complications. Here we reported a case of diagnosed endobronchial neurofibroma that was successfully resected with transbronchial electrical snaring and laser coagulation. Moreover, the relevant literature was reviewed to raise awareness of this disease.

**Patient concerns:**

A 57-year-old man presented to our hospital with cough, sputum, and shortness of breath for 2 days. Physical examination was normal. Laboratory tests revealed moderately increased C-reactive protein. Chest computed tomography showed a 10 × 8 mm round, polypoid-shaped nodule located in the left main bronchus, which was heterogeneous after contrast enhancement. It demonstrated a smooth, round, hypervascularized neoplasma obstructing most of the lumen of the upper left main bronchus under bronchoscopy.

**Interventions and outcomes:**

The tumor was removed with electrical snaring and laser coagulation completely instead of surgical resection, without any complications. Pathologically, it was confirmed of endobronchial neurofibroma. Repeated bronchoscopy showed no recurrence of the tumor, and the procedure site healed with a little of fibrotic scar formation.

**Lessons:**

Endobronchial neurofibroma is rare. Although the standard treatment for endobronchial neurofibroma is surgery, transbronchial endoscopic resection (electrical snaring and laser coagulation) is an applicable option, especially for those lesions strictly in the lumen.

## 1. Introduction

Neurogenic tumor is an autosomal dominant disease that originated from the sympathetic or intercostal nerves and is often associated with neurofibromatosis 1 (Von Recklinghausen disease).^[1]^ Intrapulmonary neurofibroma is uncommon, comprising <1% of all lung neoplasms.^[2]^ Endobronchial neurofibroma is extremely rare. The majority of endobronchial neurofibroma may remain benign and symptomatic. It has been reported that up to approximately 10% of neurofibromas may undergo malignant change.^[3]^ Although surgical resection has been the main option for the treatment of neurofibromas previously, endobronchial endoscopic intervention is widely available as a new choice of minimally invasive therapy. Herein, we present a case of endobronchial neurofibroma with respiratory symptoms and reviewed the relevant literature. The purpose of this case report was expected to improve our understanding of the endobronchial neurofibroma and its treatment strategies. This study was approved by the Institutional Ethics Board of Shaoxing People’s Hospital and the patient has provided informed consent for publication of the case.

## 2. Case presentation

**Figure 1.**
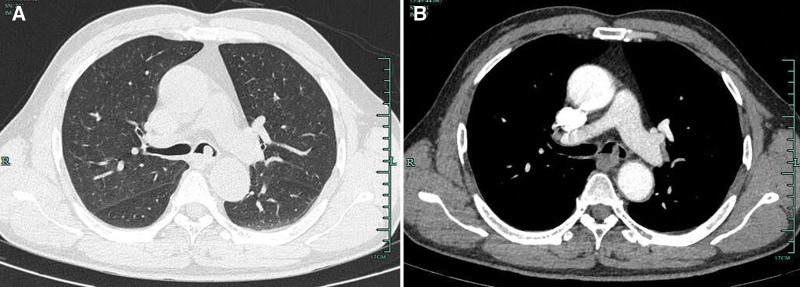
Chest CT finding of the left main bronchus nodule. (A) Lung window chest CT scanning image showed a 10 × 8 mm round, smooth-surface nodule located in left upper main bronchus. (B) Enhanced mediastinal windows show the nodule was heterogeneous contrast enhanced. CT = computed tomography.

**Figure 2.**
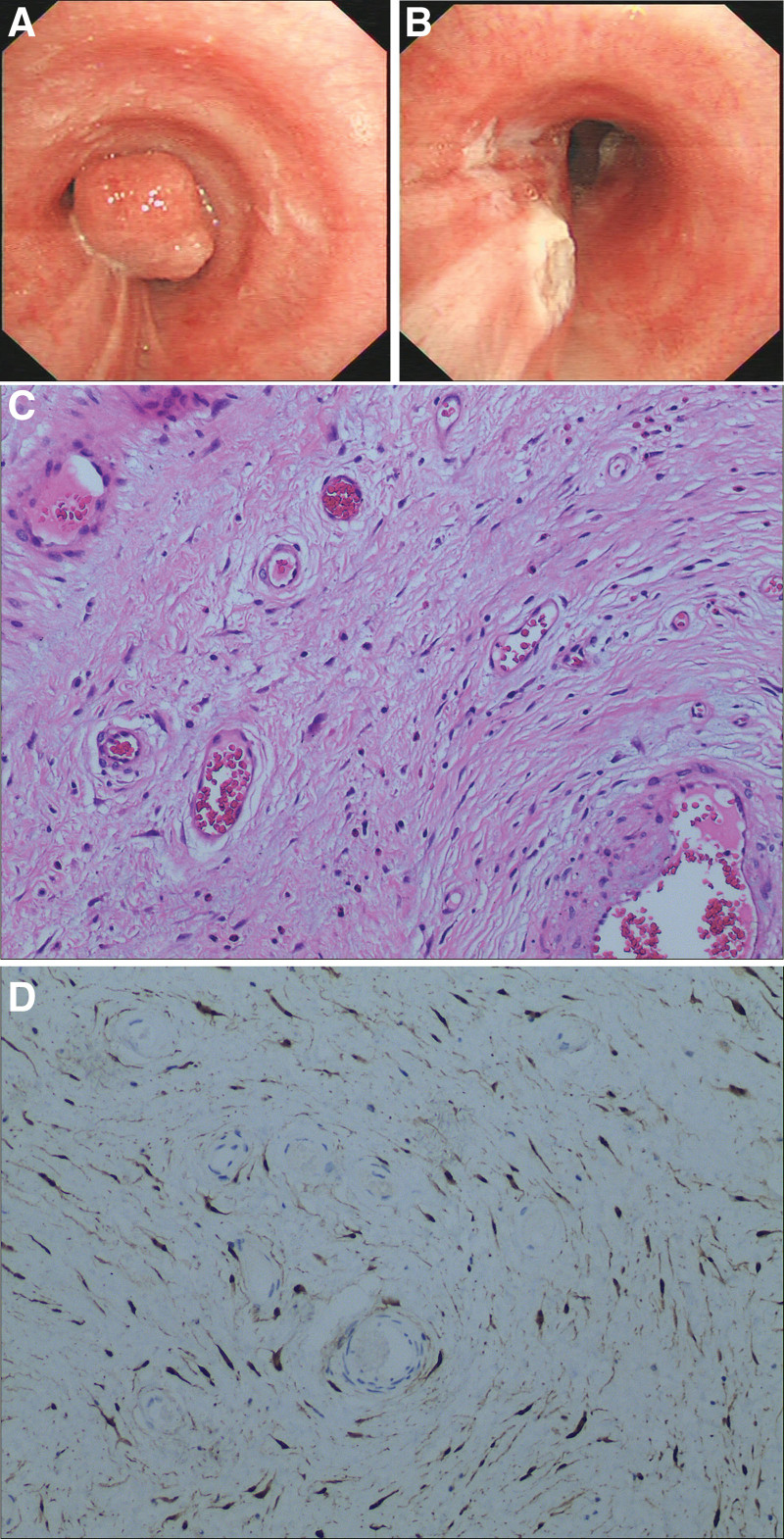
The bronchoscopy and microscopic view of the neurofibroma. (A) Bronchoscopy showed a smooth, round, hypervascularized tumor obstructing most of the lumen of the upper left main bronchus before resection. (B) The tumor was resected completely without any complications under transbronchial electrical snaring and laser coagulation. (C) Pathological section showed bundles of spindle-shaped cells arranged in strips and waves, with interstitial vascular proliferation (HE ×100). (D) The tumor cells showed positive immunohistochemical staining for S-100 protein (×100).

**Figure 3.**
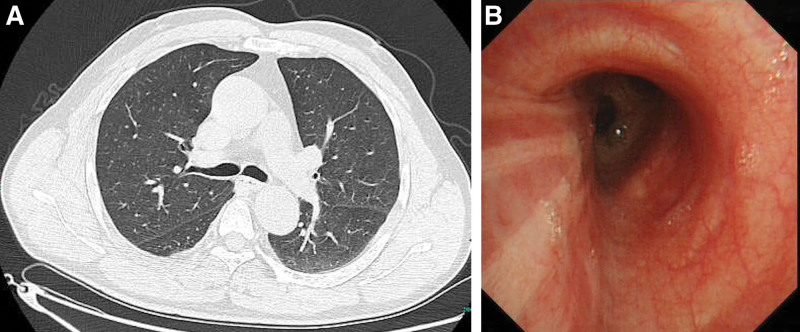
Follow-up examinations findings. (A) The left upper main bronchus was clear and no evidence of recurrence of neurofibroma on chest CT. (B) Repeated bronchoscopy showed no regrowth of the neurofibroma, and the procedure site was healed with a little of fibrotic scar formation. CT = computed tomography.

A 57-year-old man was admitted to our hospital presenting with cough, sputum, and shortness of breath for 2 days. He had been smoking and drinking for more than 40 years. His past medical and family histories were not special. Physical examination was normal. Laboratory test revealed moderate increased C-reactive protein (41.94 mg/L). The complete blood count, serum biochemistry, and serum tumor markers (carcinoembryonic antigen, squamous cell carcinoma antigen, alpha fetoprotein, carbohydrate antigen 199, and carbohydrate antigen 125) were normal. He was given ceftriaxone for 1 day before admission, but had no relief of the symptoms. Pulmonary function test revealed a forced vital capacity of 2.43 L (101% predicted), forced expiratory volume in 1 second of 2.02 L (104% predicted), forced expiratory volume in 1 second/forced vital capacity of 83.1%, and bronchodilator test was negative. Chest computed tomography (CT) showed a 10 × 8 mm round, polypoid-shaped nodule located in the left main bronchus, which was heterogeneous after contrast enhancement (Fig. 1A, B). Bronchial malignancy was initially diagnosed and he had received bronchoscopy subsequently. It demonstrated a smooth, round, hypervascularized neoplasma obstructing most of the lumen of the upper left main bronchus. We observed that the tumor had moved while breathing (Fig. 2A). Considering the tumor with a stump and narrow base, it was removed with electrical snaring, then laser coagulation was performed to prevent recurrence. The tumor was completely resected without any complications (Fig. 2B). Microscopically, the tumor was composed of waved, interlaced and spindle-shaped cells. Immunohistochemistry of the tumor was positive for S-100 protein and CD43 and negative for CKpan, Desmin, and smooth muscle actin, which confirmed the diagnosis of neurofibroma (Fig. 2C, D). The patient was discharged 2 days after transbronchial treatment with no symptoms. Follow-up examinations performed 4 months later showed no evidence of recurrence of the lesion on Chest CT scan (Fig. 3A). Repeated bronchoscopy showed there was no recurring neurofibroma, and the procedure site was healed with a little of fibrotic scar formation (Fig. 3B). Nine months after resection, the patient had no recurrent symptoms or complaints.

## 3. Discussion

Neurofibromas are usually benign that originate from nerve fiber tissue and typically arise in the peripheral nervous system of Schwann cells or neurons.^[4]^ It may occur in various parts of the whole body, including skin, skeleton, and central nervous system.^[5,6]^ The pathogenesis of the neurofibromas is multifactorial. Genetic and environmental factors may play a role in its development. Posterior mediastinum is the most common site of neurofibromas in chest, while endobronchial neurofibromas are rare. We researched literature using the databases of “Medline,” “PubMed,” or “Web of science” and using the search strategy: “Trachea,” “Bronchus,” “Bronchial,” “Endobronchial” combined with “Neurofibroma,” “Neurofibromatosis” from 2000 to 2024. A total of 12 articles and 13 cases were enrolled (Table 1).^[2,3,7–16]^

**Table 1:** Literature review of tracheal or bronchial neurofibroma.

Reference	Year	Sex/age	Underlying disease	Symptom	Physical examination	Location	Bronchoscopic examination	Complication	Treatment	Prognosis
Cranshaw et al^[7]^	2001	Female/48	NFT-1	Breathlessness	NR	Trachea	Luminal narrowing, intramural thickening	None	Multiple stents	No recurrence
Hsu et al^[3]^	2002	Male/52	None	Shortness of breath, cough	Coarse rales, decreased breath sounds	RMB	Hypervascular, round tumor with wide base	Obstructive pneumonitis	Pneumonectomy	No recurrence
Willmann et al^[8]^	2002	Female/33	NFT-1	Shortness of breath	Cutaneous neurofibromas, stridor	Upper trachea	NR	None	Surgery	No recurrence
Moorthy et al^[9]^	2005	Male/43	NFT-1, COPD, Asthma	Breathlessness	Neurofibromas	Mid-tracheal	Smooth tumor	None	Transbronchial laser excision	No recurrence
Suzuki et al^[10]^	2005	Male/67	None	Cough	NR	LULB	NR	None	Transbronchial electrical snaring, laser abrasion	NR
	2005	Male/34	None	Cough, dyspnea	NR	RMB	Smooth white-yellowish polypoid tumor	None	Transbronchial electrical snaring, laser abrasion	No recurrence
Yuan et al^[11]^	2012	Male/47	None	Cough, dyspnea, fever	Dull at percussion, coarse and moist rales	LMB	Hypervascular, smooth and spherical tumor	Obstructive pneumonia	Surgery	NR
Feng et al^[12]^	2013	Female/42	None	Shortness of breath, cough	Decreased breath sound, coarse rales	Carina, RMB, RULB	Obstructing the lumen of lower trachea	Obstructive pneumonia	Pneumonectomy	No recurrence
Venugopal et al^[13]^	2013	Female/24	Neurofibromatosis	Cough, breathlessness	NR	Both main bronchi	Multiple nodular	None	None	NR
Rabeau et al^[2]^	2016	Female/53	Chronic hepatitis C	Dyspnea, cough, sputum	Pectus excavatum	Upper trachea	Polypoid tumor moved with breathing	None	Transbronchial laser abrasion, cryotherapy	No recurrence
Srivali et al^[14]^	2016	Female/68	None	Cough, shortness of breath	None	LULB	Hypervascular, round tumor obstructing lumen	None	Thoracotomy	No recurrence
Cilleruelo Ramos et al^[15]^	2020	Male/24	NFT-1	Dyspnea	NR	Trachea	Sessile mass	None	Transbronchial resection, laser coagulation	No recurrence
Yoo et al^[16]^	2022	Female/65	Hypertension	Dyspnea, cough	Stridor	Distal trachea	Hypervascular, polypoid mass	None	Transbronchial cryotherapy	No recurrence

COPD = chronic obstructive pulmonary disease, LMB = left main bronchus; LULB = left upper lobe bronchus, NFT-1 = neurofibromatosis type 1, NR = not reported, RMB = right main bronchus, RULB = right upper lobe bronchus.

In our review of the literature, we found 7 female and 6 male patients, 4 with an underlying disease of neurofibromatosis type 1. The age ranged from 24 to 68 years (mean: 46.2). All of the patients reported in the literature were symptomatic. Cough and shortness of breath were the most common symptoms. The patients may complaint of fever and purulent sputum with obstructive pneumonia or atelectasis. Hemoptysis may be occasionally occur when a tumor is massive and hypervascular.^[17]^ A few asymptomatic endobronchial neurofibromas were detected incidentally on routine physical examination by chest imaging. This may be related to the small size and low position of the lesion.

Chest CT (especially enhanced CT) is considered to be useful for diagnosing tracheal or bronchial neurofibromas.^[8]^ CT scans can not only evaluate the size and the location of the lesions but can also identify the relationship with the surrounding blood vessels. In general, neurofibromas appear as hypodense-to-isodense on CT compared to adjacent muscle tissue.^[18]^ The lesion showed mild or moderate attenuation on enhanced CT. Bass et al^[19]^ reported that neurofibromas were heterogeneously contrast enhanced due to the variable lipid or water content within the mucinous matrix, presence of cystic degeneration, or the entrapment of perineural adipose tissue.

Bronchoscopy is effective in the diagnosis and therapy of airway tumors. Neurofibromas often present as round, smooth-surface, polypoid-shaped nodule or mass with hypervascular under bronchoscopy.^[4,20]^ We analyzed the characteristic based on the location of the lesions and found that most tumors were located in the trachea. Four cases were broad base and 3 were polypoid type. As in our case, the tumor was in the left main bronchus with smooth, round, obstructing the lumen. Furthermore, it moved with breathing owing to its narrow base.

The treatment of endobronchial neurofibroma remains controversial. Although surgical resection is the standard therapy, endoscopic intervention has been involved gradually.^[15,16]^ In our literature review, 5 cases were treated with surgical resection, and 7 were treated with transbronchial therapy with stents, laser, electrical snaring, or cryotherapy. All of the patients had a good prognosis except 3 that were not reported. We combined the literature with our clinical practice, and we believe that the strategy for endotracheal-bronchial neurofibromas depends on the size and the location of the lesions. For polypoid tumors as well as narrow base that are strictly in the lumen, endobronchial treatment should be the first choice.^[14]^ However, endoscopic intervention is not applicable when the tumors invade adjacent tissues or result in complications such as obstructive pneumonitis and atelectasis.^[12]^ In addition, surgical resection is recommended for the following conditions^[3,8,11,12,14]^: difficulty in making diagnosis or a possible malignancy; evidence of invasion of adjacent structures or destruction of peripheral lung tissue; complications such as uncontrolled hemoptysis or the tumor remaining after transbronchial resection; and anticipated technical difficulties for bronchoscopic treatment.

In conclusion, endobronchial neurofibroma is a rare benign neurogenic tumor that is easily misdiagnosed due to atypical clinical symptoms. Transbronchial endoscopic intervention is recommended when the lesion is narrow base or polypoid, which is strictly located in the lumen without other complications.

## Acknowledgments

The authors would like to thank Professor Yinan Yao for performing language editing.

## Author contributions

**Conceptualization:** Xing Chen.

**Writing – original draft:** Xing Chen.

**Writing – review & editing:** Xing Chen, Shuying Liu.

**Data curation:** Shanlu Yu.

**Formal analysis:** Jian Sun.

**Project administration:** Jian Sun.

**Methodology:** Yefeng Chen.

**Investigation:** Chunyi Zhang.

**Supervision:** Hua Wang, Min Xiang.
